# Pulmonary delivery of the broad-spectrum matrix metalloproteinase inhibitor marimastat diminishes multiwalled carbon nanotube-induced circulating bioactivity without reducing pulmonary inflammation

**DOI:** 10.1186/s12989-021-00427-w

**Published:** 2021-09-08

**Authors:** Tamara L. Young, Ekaterina Mostovenko, Jesse L. Denson, Jessica G. Begay, Selita N. Lucas, Guy Herbert, Katherine Zychowski, Russell Hunter, Raul Salazar, Ting Wang, Kelly Fraser, Aaron Erdely, Andrew K. Ottens, Matthew J. Campen

**Affiliations:** 1 Department of Pharmaceutical Sciences, MSC09 5360, 1 University of New Mexico, Albuquerque, NM 87131-0001 USA; 2 Department of Anatomy and Neurobiology, Virginia Commonwealth University, PO Box 980709, Richmond, VA 23298 USA; 3 College of Nursing, University of New Mexico, Albuquerque, NM 87131 USA; 4 Department of Internal Medicine, University of Arizona College of Medicine, Phoenix, AZ USA; 5 Pathology and Physiology Research Branch, National Institute for Occupational Safety and Health, Morgantown, WV 26505 USA

**Keywords:** Multiwall carbon nanotube (MWCNT), Carbon nanotubes, Nanoparticles, Nanomaterials, Matrix metalloproteinase, Inflammation, Serum-bioactivity, Biomarker

## Abstract

**Background:**

Multiwalled carbon nanotubes (MWCNT) are an increasingly utilized engineered nanomaterial that pose the potential for significant risk of exposure-related health outcomes. The mechanism(s) underlying MWCNT-induced toxicity to extrapulmonary sites are still being defined. MWCNT-induced serum-borne bioactivity appears to dysregulate systemic endothelial cell function. The serum compositional changes after MWCNT exposure have been identified as a surge of fragmented endogenous peptides, likely derived from matrix metalloproteinase (MMP) activity. In the present study, we utilize a broad-spectrum MMP inhibitor, Marimastat, along with a previously described oropharyngeal aspiration model of MWCNT administration to investigate the role of MMPs in MWCNT-derived serum peptide generation and endothelial bioactivity.

**Results:**

C57BL/6 mice were treated with Marimastat or vehicle by oropharyngeal aspiration 1 h prior to MWCNT treatment. Pulmonary neutrophil infiltration and total bronchoalveolar lavage fluid protein increased independent of MMP blockade. The lung cytokine profile similarly increased following MWCNT exposure for major inflammatory markers (IL-1β, IL-6, and TNF-α), with minimal impact from MMP inhibition. However, serum peptidomic analysis revealed differential peptide compositional profiles, with MMP blockade abrogating MWCNT-derived serum peptide fragments. The serum, in turn, exhibited differential potency in terms of inflammatory bioactivity when incubated with primary murine cerebrovascular endothelial cells. Serum from MWCNT-treated mice led to inflammatory responses in endothelial cells that were significantly blunted with serum from Marimastat-treated mice.

**Conclusions:**

Thus, MWCNT exposure induced pulmonary inflammation that was largely independent of MMP activity but generated circulating bioactive peptides through predominantly MMP-dependent pathways. This MWCNT-induced lung-derived bioactivity caused pathological consequences of endothelial inflammation and barrier disruption.

## Introduction

Engineered nanomaterials (ENM; materials with at least one dimension < 100 nm) represent one of the fastest growing sources of technological advances over the last two decades. Significant government and private investments to the tune of an estimated US$8.5 billion global market value was reported in 2019 and this is expected to reach US$9.6 billion in 2020 [1]. Optimism for the future of ENM application is tempered by concerns regarding both potential human health effects and environmental impacts of production and use [1–6]. Carbon nanotubes (CNT), consisting of single-walled (SWCNT) and multi-walled (MWCNT) varieties, represent one of the fastest growing ENM fields. The unique physical, chemical, and electrical properties make CNT suitable for a wide range of applications and accounts for their popularity [7, 8]. As the body of literature around CNT toxicity increases, common findings of inhalation exposure to CNT include disruption of lung tissue architecture, recruitment of inflammatory cells and activation of inflammatory responses [9–13].

The extracellular matrix (ECM), which plays a significant role in these processes, is a complex dynamic structure ubiquitous in all tissues and is in a perpetual state of controlled degradation and regeneration [14]. Uncontrolled degradation of the ECM can lead to homeostatic dysregulation and pathologic transformation. The process of ECM turnover is facilitated mainly by matrix metalloproteinases (MMPs), a large family of zinc-dependent endopeptidases [15–17]. ECM degradation generates bioactive peptide fragments termed “matrikines” that function in a wide array of biological processes to facilitate tissue homeostasis, signal transduction and wound healing [18]. Within the lung, ECM degradation products have been implicated in pathologic processes leading to fibrotic transformation and chronic obstructive pulmonary disease (COPD) [19]. The tripeptide fragment pro-gly-pro (PGP), an ECM collagen derived neutrophil chemokine containing sequences similar to those that bind the CXCR1/2 receptors [20], has been proposed as a biomarker in COPD and in cystic fibrosis pathogenesis [21, 22]. PGP is generated in a feedback loop following MMP-8 and MMP-9 activation and is implicated in sustained neutrophilic inflammation [22–25].

We previously demonstrated a link between ENM exposure, specifically MWCNT, and MMP activity. Wild-type mice exposed to MWCNT exhibited serum bioactivity that led to dysregulated endothelial function in treated vessels ex vivo; this bioactivity was largely absent in serum from MMP-9 knockout mice exposed to MWCNT [26]. Serum from thrombospondin (TSP), a matrix glycoprotein and MMP-9 substrate, knockout animals failed to impair vasodilation in naive vessels [27]. As we recently demonstrated, MWCNT exposure produced serum bioactivity that resulted in blood–brain barrier disruption and neuroinflammatory activation; a TSP peptide fragment was a key player in this outcome [28]. These findings collectively implicate proteinase activity in the lung as an indirect driver of systemic vascular outcomes. The specific mechanisms underlying pulmonary-derived serum bioactivity is still unclear. However, serum peptidomic analysis indicated significantly increased lung protease expression, including overlapping and independent peptide profiles between the lung and systemic circulation [29]. These peptide profiles included significant changes in MMP substrates with functionality in ECM organization, inflammation, cardiovascular pathology, cell receptor signaling and exosome components [29]. Understanding the role of MMPs in generating ECM-derived matrikines (and many other peptide fragments) may represent novel biomarkers of exposure—and potentially drivers of morbidity—not previously explored. In this study, we investigated the role of MMPs in MWCNT-exposure derived serum bioactivity by utilizing a broad-spectrum MMP inhibitor delivered selectively to the lung.

## Methods

### Animals and exposures

Specific pathogen-free 6–8-week-old male C57BL/6J mice (Jackson Laboratory) were used in this study. Animals were housed in an Association for Assessment and Accreditation of Lab Animal Care International-approved animal facility at the University of New Mexico with procedures approved by Institutional Animal Care and Use Committee of the University of New Mexico. Animal care and use procedures were conducted in accordance with the US Public Health Service’s Policy on Humane Care and Use of Laboratory Animals (https://grants.nih.gov/grants/olaw/references/phspol.htm) and the National Institutes of Health’s Guide for the Care and Use of Laboratory Animals (https://grants.nih.gov/grants/olaw/Guide-for-the-Care-and-Use-of-Laboratory-Animals.pdf). Normal chow and water were provided ad libitum in ventilated cages in a temperature- and humidity-controlled environment with a 12-h light/dark cycle.

**Fig. 1:**
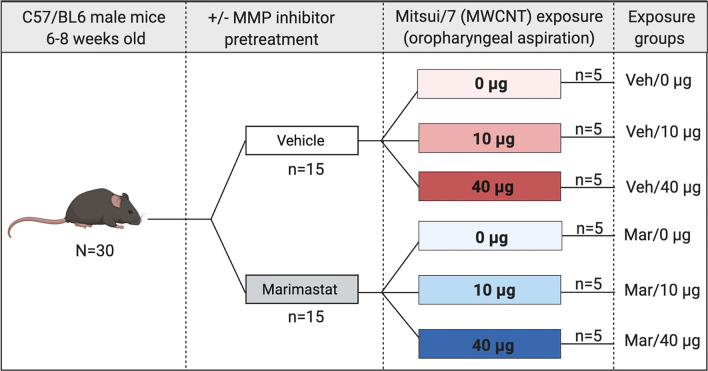
Experimental Design. Male C57BL/6 mice aged 6–8 weeks were randomized into 6 groups of 5 mice each. Animals were weighed and dosed with 10 mg/kg body weight Marimastat via oropharyngeal aspiration 1 h prior to dosing with dispersion media (DM; 0 µg), 10 µg, or 40 µg of MWCNT via oropharyngeal aspiration. Animals were euthanized 24-h post MWCNT exposure and tissues collected to assess pulmonary inflammation, serum peptide profile and serum bioactivity. (Figure generated in BioRender)

**Fig. 2:**
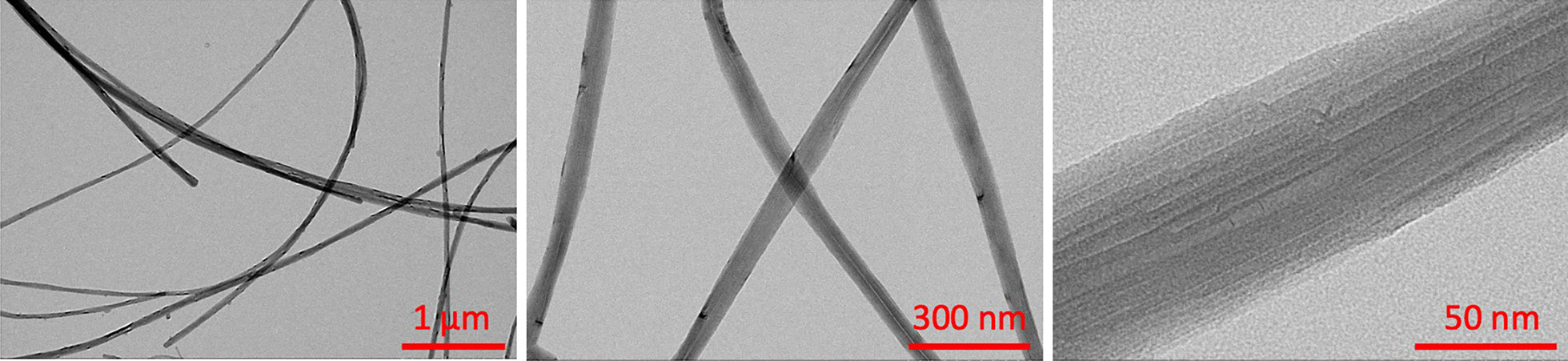
Electron microscopy images of the Mitsui-7 MWCNT used for this study, at increasing magnification

The C57BL/6 J mice were randomized into six groups and treated via oropharyngeal aspiration under light isoflurane anesthesia. A broad-spectrum matrix metalloproteinase (MMP) inhibitor, Marimastat (Alfa Aesar—J67288) dissolved in 1% Dimethyl Sulfoxide (DMSO) at a final concentration of 10 mg/kg of body weight, or 1% DMSO in 1× phosphate buffered saline (PBS; Fig. 1) was administered to half of the animals 1 h (h) prior to exposure to multiwalled carbon nanotubes (MWCNT). MWCNTs were prepared in dispersion media (DM) consisting of mouse serum albumin (0.6 mg/mL) and 1,2-dipalmitoyl-sn-glycero-3-phosphocholine (10 μg/mL), sonicated prior to administration and vortexed between each animal dosed. The MWCNT material used in this study, MWCNT-7, has been extensively characterized [30–34]. MWCNT were examined by transmission electron microscopy (TEM) on a JEOL2100F TEM (JEOL USA, Inc., Peabody, MA, USA) using the modified NMAM for CNT on MCE Filters by TEM as previously described [34, 35]. The average diameter between studies ranges from 49 to 63 nm with an average length around 4 μm (Fig. 2). Purity was > 99% carbon. MWCNT was administered at doses of 0 μg (DM only), 10 μg or 40 μg MWCNT suspended in 50 µl volume (n = 5/grp) (Fig. 1); doses were based on prior findings [26, 29] to permit the current mechanistic follow up study. Mice were euthanized at 24 h following MWCNT pulmonary dosing using isoflurane and exsanguination. At the time of euthanasia, blood and bronchoalveolar lavage fluid (BALF) were collected; blood was allowed to clot on ice and then centrifuged to collect serum. Following transcardial perfusion with ice-cold 1× PBS, lavaged lungs and heart were collected and flash frozen in liquid nitrogen for later determination of changes in relative mRNA expression.

### Pulmonary inflammation assessment

#### Bronchoalveolar lavage fluid (BALF) Cell composition

Lung lavage fluid was collected by instilling 1 mL of sterile PBS and immediately withdrawing fluid twice via cannulated trachea. Cell counts were determined by centrifuging BALF, less 200 μL, at 1800×*g* for 5 min to form a cell pellet. The pellet was re-suspended, stained with trypan blue for a viable cell count with a hemocytometer. Cells were seeded at 10,000 cells/slide and the total cells were manually counted under a light microscope. Differential BALF cell counts were assessed by centrifuging 200 μL of BALF onto a cytospin slide preparation and the number of polymorphonuclear neutrophils and macrophages quantified using HEMA3 stain (Protocol, Thermo Fisher Scientific).

#### BALF cytokine levels

MWCNT-induced cytokine protein changes in BALF were determined using electrochemiluminescence. The Meso Scale Discovery MULTI-SPOT V-PLEX® Cytokine Assay System Proinflammatory Panel 1 (mouse) kit (K15048D—Meso Scale Diagnostics LLC, Rockville, MD) was used according to manufacturer’s instructions. Briefly, 50 μL/well of the BALF supernatant were loaded onto sample plates pre-coated with capture antibodies for the following cytokines: IL-1β (interleukin 1 beta), IL-2 (interleukin 2), IL-4 (interleukin 4), IL-5 (interleukin 5), IL-6 (interleukin 6), IL-10 (interleukin 10), IL-12p70 (interleukin 12 active heterodimer), IFN-γ (interferon gamma), TNF-α (tumor necrosis factor alpha) and KC/GRO (CXCL1, chemokine C-X-C motif ligand 1). Plates were incubated with gentle shaking for 2 h at room temperature. Plates were washed 3× with buffer containing 1× PBS and 0.05% Tween 20. Detection antibody was added to each well and allowed to react for 1 h at room temperature under gentle shaking. Plates were washed as above, and Read Buffer was added to each well. Plates were analyzed on an MSD QuickPlex SQ 120 instrument (MSD, AI0AA-0); Discovery Workbench (v. 4.0) software calculated cytokine concentrations using a linear regression analysis of the standard curve. All concentrations were normalized to total BALF protein, determined using a standard Bradford protein assay.

#### Whole lung cytokine gene expression analysis

Flash frozen lung tissue was homogenized in lysis buffer (Buffer RLT) and total RNA was isolated according to the RNeasy Mini Kit (QIAGEN, Germantown, MD) protocol. RNA (1 µg) was reverse transcribed using High-Capacity cDNA Reverse Transcription Kit (Applied Biosystems; 4368814) in a thermal cycler under the following conditions: Step 1: 25 °C for 10 min; Step 2: 37 °C for 120 min; Step 3: 85 °C for 5 min; and Step 4: 4 °C for infinity. Generated cDNA (50 ng) was used to assess gene expression changes via quantitative real-time PCR (qPCR) in a 96-well format. Expression of murine *Il1β* (Interleukin 1 beta; Mm00434228_m1), *Il6* (Interleukin 6; Mm00446190-m1), *Ccl2* (C–C Motif Chemokine Ligand-2; Mm00441242_m1), *Tgfb* (Transforming growth factor beta 1; Mm01178820_m1)*, Tnfα* (Tumor necrosis factor alpha; Mm00443258_m1), *Icam1* (Intercellular adhesion molecule 1; Mm00516023_m1), and *Vcam1* (Vascular cell adhesion molecule 1; Mm01320970_m1) (Applied Biosystems, Foster City, CA) were measured using the TaqManR Gene Expression protocol (ThermoScientific, Waltham, MA) following the manufacturer’s instructions. Relative gene expression normalized to the housekeeping gene TATA-Box Binding Protein (*tbp*; Mm00446973_m1) was determined using the 2^−ΔΔCT^ method for all samples with threshold cycle values (CT) under 35. Results are expressed as fold change.

### Serum peptidomic mass spectrometry

Endogenous peptidomic analysis was performed on mouse serum specimens collected following treatment with 0 µg, 10 µg or 40 µg of MWCNT, with and without Marimastat pretreatment (n = 5/grp) using previously described methodology [29]. Briefly, 40 µL of serum was pre-filtered using a 0.22 µm Ultrafree-MC unit (Millipore) using the manufacturer’s instructions. Samples were reduced in 18 mM TCEP and denatured in 20% acetonitrile and thiol-protection with 30 mM iodoacetamide to dissociate peptide content from carrier moieties. Samples were size-fractionated using YM-30 MicroCon units (Millipore) per manufacturer’s instructions with an effective mass cutoff of approximately 8 kDa. The retentate was further dissociated by acidification with 0.4% formic acid to disrupt ionic interactions and re-spun through the YM-30 to combine filtrates. The filtrate was de-salted, de-lipidated and concentrated by solid-phase extraction using a Symmetry C18 reversed-phase column (Waters). The enriched peptide samples (4 µL) were then gradient separated (6–44% acetonitrile in 0.1% formic acid) using a 150 mm × 75 µm HSS T3 reversed-phase capillary column on a NanoAcquity UPLC online with a Waters Synapt G2-Si tandem mass spectrometer (Waters). The instrument was operated in UDMSe mode [36] for data-independent analysis at 25,000 resolving power and the quadrupole optimized to exclude small-molecule ions below 500 m/z. UDMSe data were peak picked 150 and 50 ion count thresholds for low-energy and high-energy scans, respectively, and deisotoped using ProteinLynx Global SERVER™ (PLGS) software v3.0.3 (Waters). Resultant ion tables were aligned across replicates by retention time (± 1.5 min), drift time (± 5 bins) and accurate mass (± 12 ppm) measures with EndogeSeq (https://ottenslab.weebly.com/endogeseq.html). Precursor ion tables were then filtered to retain only highly reproducible measures (n ≥ 4/grp), with matched product ions (± 24 ppm) retained if present in at least two biological replicates. Left censored data were imputed by random-generated value sets centered at the limit of quantification accounting for the datasets median ion variance and missingness across replicates [37]. The compiled peptidomic data were median centered and log_2_ transformed before testing for effects of MWCNT exposure, Marimastat pretreatment or their interaction using 2-way Analysis of Variance (ANOVA) and Benjamini–Hochberg multiple-measures correction in Multiple Experiment Viewer (mev.tm4.org), set to a 5% false discovery rate.

### Serum cumulative inflammatory potential assay

Mouse cerebrovascular endothelial cells (mCECs) were obtained from a commercial vendor (Cell Biologics, Chicago, IL) and maintained according to manufacturer’s recommendations at 37 °C and 5% CO_2_ with complete endothelial cell medium supplemented with 5% fetal bovine serum. All experiments were conducted with cells between passages 3 and 8. To determine the serum cumulative inflammatory potential of MWCNT exposure, mCECs were treated with serum isolated from exposed or control C57BL/6 male mice as previously described [38, 39]. Briefly, mCECs were serum starved overnight then incubated in FBS-free culture media supplemented at a final concentration of 5% v/v serum from dispersion media control (0 μg; DM), 10 μg, or 40 μg MWCNT-exposed mice for 4 h. RNA was isolated from treated mCECs using the RNeasy Mini Kit (QIAGEN, Germantown, MD), and 1 µg was reverse transcribed prior to gene expression analyses via quantitative real-time PCR (qPCR). Furthermore, in an additional permutation, cells were pretreated with either non-immune IgG or a cluster of differentiation 36 (CD36) neutralizing antibody to test the role of CD36 in mediating responses (1 µg/mL, 30 min prior to serum). Expression of mouse *Ccl2, Il6, Tgf-β, Icam1*, and *Vcam1* were measured, as described above. Relative gene expression, normalized to the housekeeping gene *Tbp* was determined using the 2^−ΔΔCT^ method for all samples with cycle threshold (CT) values under 35. Results are expressed as fold change.

### Monolayer impedance-based quantification of cell growth and barrier integrity

Electric Cell-substrate Impedance Sensing (ECIS; Applied Biophysics) system was used to evaluate endothelial barrier integrity changes of mCECs in a monolayer following exposure to serum from DM- or MWCNT-exposed mice. Briefly, cells were grown in ECIS culture chambers on top of an opposing circular gold electrode array. A constant small alternating current was applied between the electrodes and the changes in electrical potential across the monolayer was measured and resistance changes were recorded every minute. mCECs were allowed to come to confluence, then serum-starved overnight. The following day, serum-free culture media was supplemented with serum from DM- or MWCNT-exposed mice at a concentration of 5% v/v. The cells were then incubated at 37 °C and 5% CO_2_ for 4 h. Baseline serum-response readings were captured over this period and then an electric current was passed through the gold electrode to disrupt the confluent cell layer (“wounding”). The rate of recovery from wounding was evaluated by assessing the length of time for resistance readings to return to the pre-wounding baselines.

### Statistics

All statistics were conducted in GraphPad Prism (v9.0). Data were tested for Guassian normality using a Shapiro–Wilk test; most data sets were observed ot be normal. The infrequent nature of non-Guassian distribution of data led to the choice of standard parametric statistical tests for all assays. Data were either assessed as a 2-way ANOVA, considering MWCNT and Marimastat treatments as the 2 factors. Tukey’s post-hoc multiple comparison test was used when appropriate. For serum cumulative inflammatory potential assays, a 3-way ANOVA was conducted, including the use of IgG vs CD36 antibody treatment.

## Results

### Pulmonary inflammation

#### BALF inflammatory cell profile

**Fig. 3:**
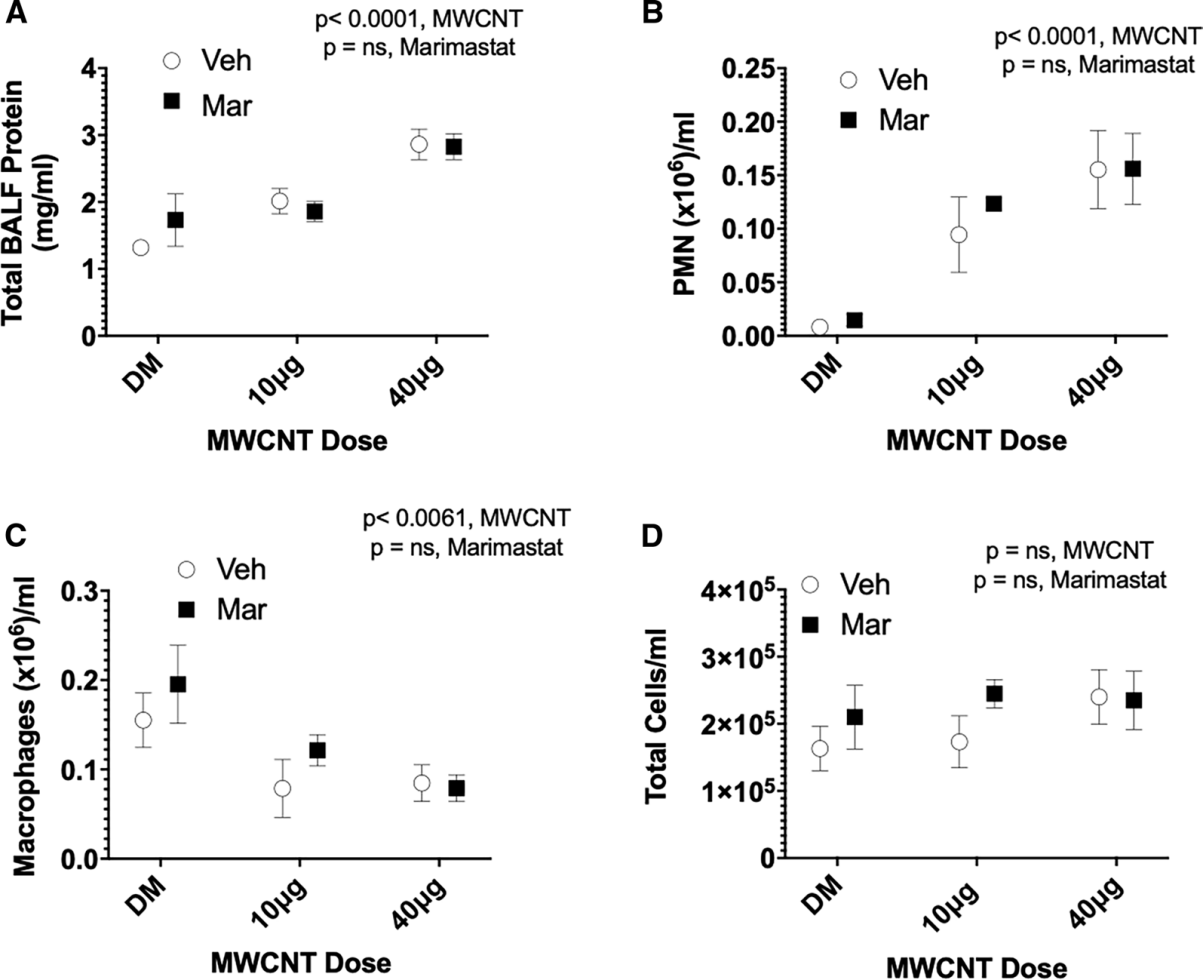
MWCNT-mediated lung inflammation. Bronchoalveolar lavage fluid (BALF) total protein and infiltrating inflammatory cell count. MWCNT exposure-induced pulmonary inflammation was not altered by MMP blockade. **a** Total lavage fluid protein concentration assessed via Bradford protein assay. MWCNT-exposure increased BALF total protein in a dose-dependent manner when compared with controls. **b** and **c** BALF cell differentials; Polymorphonuclear neutrophils (PMN) cells were significantly increased by MWCNT exposure at both low and high doses; Macrophages in BALF were decreased in MWCNT treated cells. **d** Total BALF cells were unchanged by MWCNT exposure. *p* values based on 2-way ANOVA presented for each figure. N = 4–5 per group. Data presented are means ± SEM

MWCNT-exposure drives lung inflammation via mechanisms that are predominantly independent of MMP activation. MWCNT-induced pulmonary inflammation was assessed by total protein and differential cell counts from bronchoalveolar lavage fluid (BALF) collected from DM- and Marimastat-pretreated, MWCNT-exposed mice. MWCNT induced a significant (*p* < 0.0001) dose-dependent increase in BALF total protein (Fig. 3a). Accompanying this increase was a significant influx of polymorphonuclear neutrophils (Fig. 3b). A modest reduction of macrophages was also observed (Fig. 3c), but overall, the total cell counts were stable, if not increasing in the MWCNT-exposed mice (Fig. 3d). These indicators of lung inflammatory activation occurred via mechanisms independent of MMP activity, as Marimastat pretreatment did not alter MWCNT-mediated responses.

#### BALF cytokine expression

**Fig. 4:**
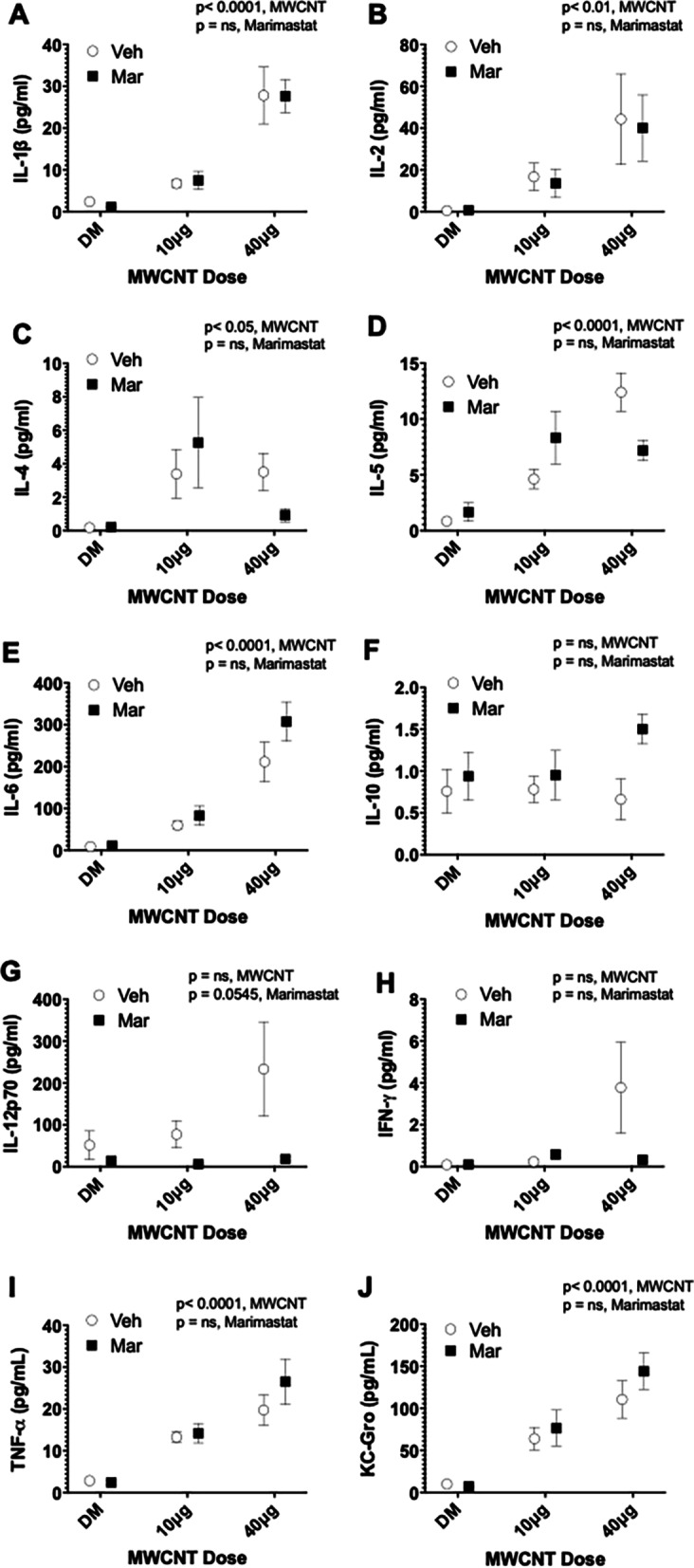
MWCNT-mediated pulmonary inflammatory activation. Bronchoalveolar lavage fluid (BALF) inflammatory cytokine profile assessed via Meso Scale Discovery multiplex cytokine assay. MWCNT-exposure increased BALF cytokine protein expression in a dose-dependent manner which was not significantly altered by MMP blockade. **a** IL-1β cytokine protein expression. **b** IL-2 cytokine protein expression. **c** IL-4 cytokine protein expression. **d** IL-5 cytokine protein expression. **e** IL-6 cytokine protein expression. **f** IL-10 cytokine protein expression. **g** IL12p70 cytokine protein expression. **h** IFN-γ cytokine protein expression. **i** TNF-α cytokine protein expression. **j** KC-Gro/CXCL1/GROα chemokine protein expression. *p* values based on 2-way ANOVA presented for each figure. N = 4–5 per group. Data presented are means ± SEM

To further investigate the role of MWCNT in the lung, we assessed cytokines in the BALF using a multiplex electrochemiluminescence assay. Most conventional inflammatory cytokines (IL-1β, IL-2, IL-4, IL-6, TNF-α, and KC-GRO) (Fig. 4a–c, e, i, j) increased in response to MWCNT treatment and were not affected by MMP inhibition. Some cytokines remained unchanged by MWCNT treatment or MMP blockade (IL-10, IL12p70 and IFNγ; Fig. 4f–h). Interestingly, IL-5 did show inhibition of MWCNT-mediated induction by Marimastat (Fig. 4d) and IL-12p70 was just outside the range of significance (*p* = 0.054) for an MMP inhibition effect (Fig. 4g). These outcomes suggest that certain cytokine induction may be downstream of inflammatory MMP induction, though most are not.

#### Whole lung cytokine gene expression

**Fig. 5:**
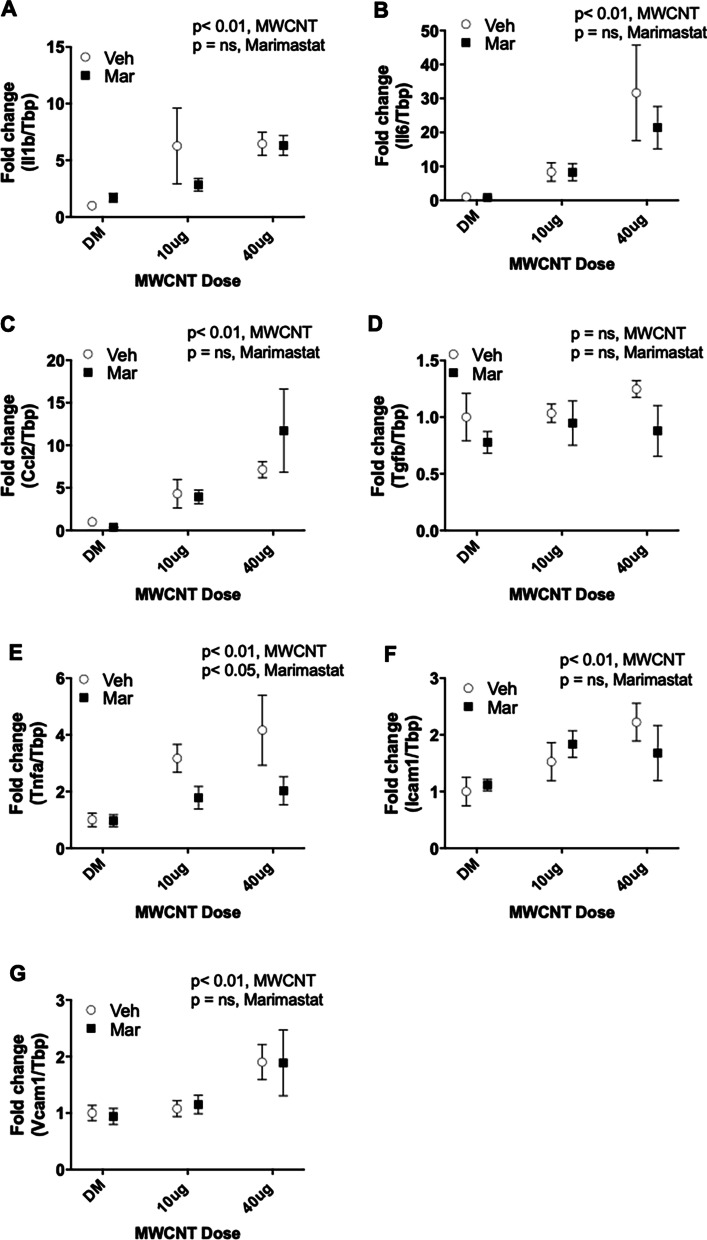
Lung cytokine gene expression profile. Cytokine expression in response to MWCNT was largely unaltered by lung MMP blockade. **a** *Il1b* gene expression. **b** *Il6* gene expression. **c** *Ccl2* gene expression. **d** *Tgfb* gene expression. **e** *Tnfa* gene expression. **f** *Icam1* gene expression. **g** *Vcam1* gene expression. Marimistat only inhibited Tnfa expression (**e**). *p* values based on 2-way ANOVA presented for each figure. N = 4–5 per group. Data presented are means ± SEM

Finally, we examined pulmonary inflammation in terms of transcriptional changes in whole lung homogenates. Most cytokine gene expression trends were consistent with protein levels in BALF, with *Il1β, Il6, Ccl2, Tnfα and Vcam1* (Fig. 5a–c, e and g) increasing in a dose-dependent manner following MWCNT treatment. Apart from *Tnfα*, which showed significant decrease at bot doses of MWCNT, this increase was unaltered by MMP inhibition. We additionally tested *Icam1* mRNA (Fig. 5f) expression as a marker of vascular injury and it showed a non-significant elevation following MWCNT exposure. Combined with BALF findings, these data show pulmonary inflammation due to MWCNT exposure is only minimally influenced by local MMP activity. Neither MWCNT nor Marimastat treatment altered *Tgf*β expression (Fig. 5d).

### MWCNT- and MMP-blockade induced serum peptide changes

**Fig. 6:**
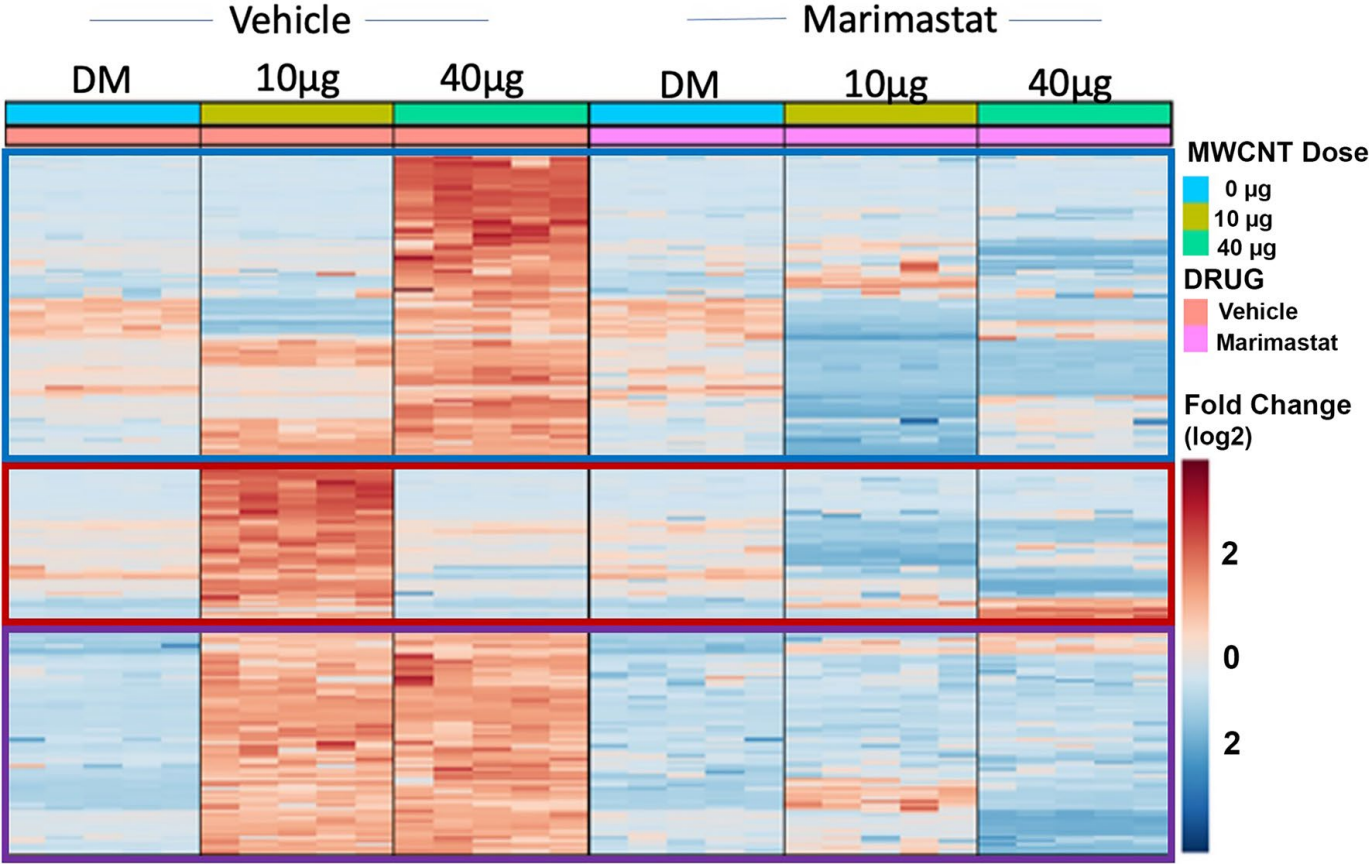
Serum peptidomics heatmap. MWCNT-induced peptide serum profile changes assessed via Mass Spectrometry. MWCNT exposure increased total serum peptide composition in a differential dose-dependent manner. Left panel: Serum peptide changes resulting from MWCNT exposure. Blue box: Serum peptide composition induced exclusively by high dose (40 µg) MWCNT exposure; Red box: Serum peptide composition induced by exclusively low dose (10 µg) MWCNT exposure; Purple box: Serum peptide composition induced by both low and high dose MWCNT exposure. Right panel: Serum peptide profile generated by MWCNT and Marimastat exposure. MWCNT-induced serum peptide changes were abrogated by broad spectrum MMP inhibitor, Marimastat. These findings indicate an MMP-mediated mechanism of action for MWCNT in generating lung-derived serum bioactivity. N = 5 per group

Consistent with recent studies of serum peptidomic changes after MWCNT exposure [29, 40], the 10 µg and 40 µg doses of MWCNT led to substantial generation of numerous circulating peptide fragments (< 8 kDa cutoff). Both doses of MWCNT produced clustering of common elevated peptides (Fig. 6, purple box), as well as induction of unique dose specific peptides (Fig. 6, 10 µg; red box, 40 µg; blue box); findings appeared consistent with more extensive characterizations of this exposure [28, 29, 41]. Importantly, pulmonary Marimastat pretreatment returned serum peptidomic profiles to near control levels by almost completely abolishing those induced peptides. Given the distinct observable changes in serum peptide profile following targeted pulmonary MMP blockade, these findings strongly suggest the serum peptides originated from MMP activation and activity in the lung following MWCNT exposure.

### Serum cumulative inflammatory potential assessment

**Fig. 7:**
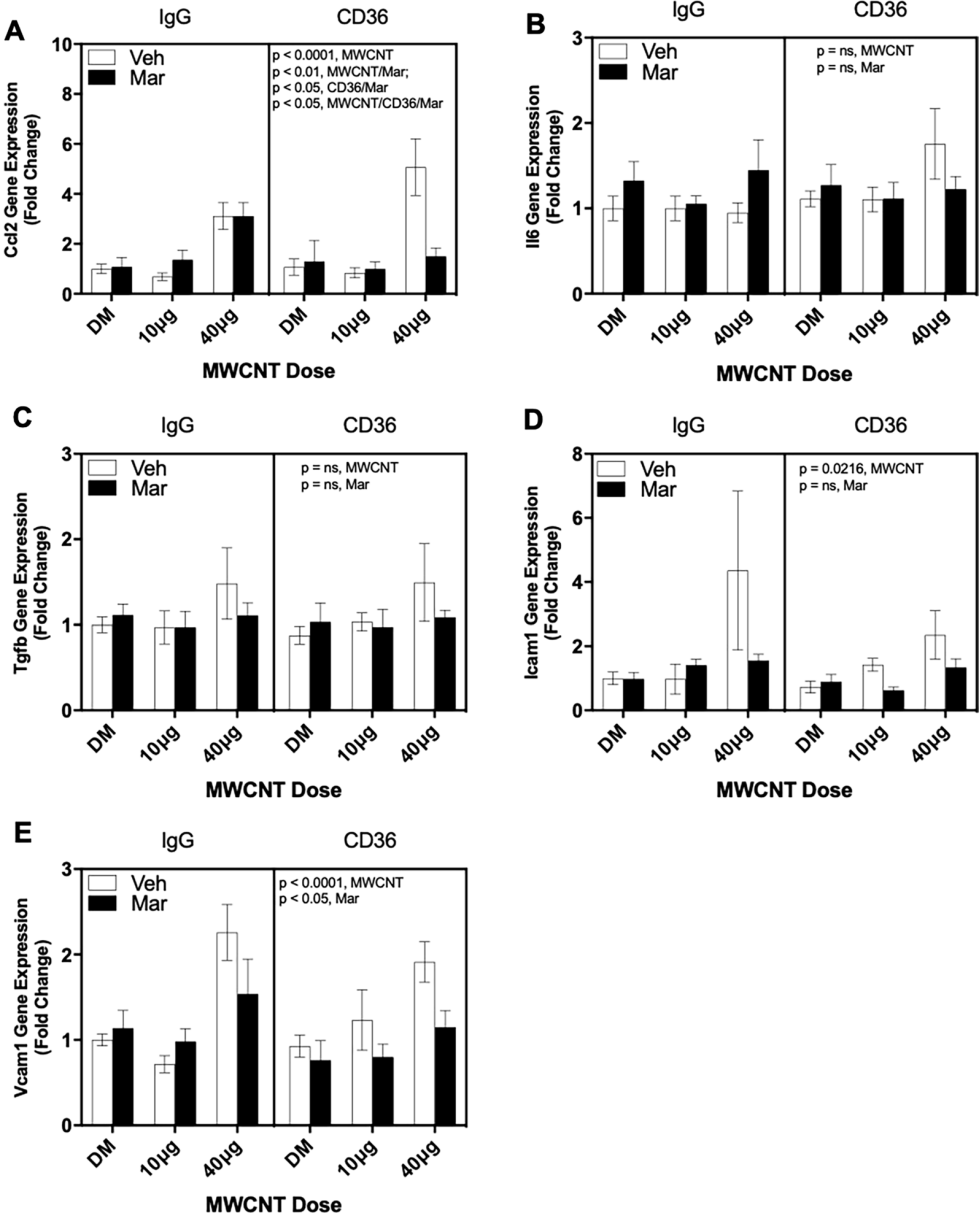
MWCNT in vitro serum bioactivity evaluation. Contribution of CD36 to MWCNT-mediated serum cumulative inflammatory potential in mouse cerebrovascular endothelial cells (mCEC). Cells in monolayer were treated with a CD36 blocking antibody or IgG control antibody, followed by 5% v/v serum from C57BL/6 mice exposed to MWCNT in conjunction with MMP blockade. qPCR performed on cell isolates to access cytokine gene expression changes. Shown are gene expression for **a** *Ccl2*, **b** *Il6*, **c** *Tgfb*, **d** *Icam1*, and **e** *Vcam1*. *p* values based on 3-way ANOVA presented for each figure. N = 4–5 per group. Data presented are means ± SEM

To determine the inflammatory bioactivity of the serum following MWCNT dosing and Marimastat pretreatment, serum (5% v/v) from control and exposed mice was added to confluent mCECs for 4 h, followed by qPCR assessment of canonical inflammatory response genes from the mCECs including *Ccl2, Il6, Tgfb, Icam1,* and *Vcam1* (Fig. 7). Additionally, since previous studies showed a potential role for CD36 in mediating serum-induced vascular reactivity changes [26], we assessed its role in vitro using an anti-CD36 antibody compared to a non-immune immunoglobulin G (IgG) control pretreatment. Three-way ANOVA analyses were therefore used to test independent or interactive roles of MWCNT, Marimastat, and CD36. MWCNT serum exposure resulted in significant upregulation of endothelial *Ccl2* and *Vcam1* mRNA (*p* < 0.0001) in both IgG and CD36 antibody treatment groups. *Ccl2* expression was reduced in the CD36 ab + Marimastat group, based on significant interaction terms in the 3-way ANOVA (*p* < 0.05). *Vcam1* expression, also significantly induced (*p* < 0.0001) by MWCNT-treated serum, was not upregulated in serum from mice treated with MWCNT and Marimastat (*p* = 0.044). *Icam1* gene expression was increased by serum from MWCNT-treated mice (*p* = 0.0216) with an apparent abrogation of this effect by Marimastat (Fig. 7d).

### Functional assessment of MWCNT-mediated serum bioactivity

**Fig. 8:**
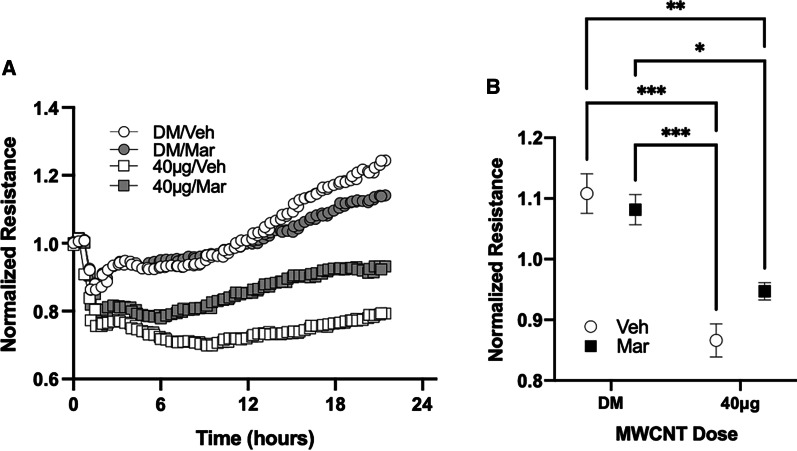
Evaluation of the role of MWCNT-exposure generated serum bioactivity on brain endothelial barrier integrity. Mouse cerebrovascular endothelial cells (MCEC) grown in a confluent monolayer were treated with serum (5% v/v) from C57BL/6 mice exposed to MWCNT in conjunction with Marimastat (Mar) or Vehicle (Veh). Barrier integrity changes were accessed via Electric Cell-substrate Impedance Sensing (ECIS). **a** Normalized electric resistance changes across endothelial monolayer were recorded over the course of 24 h. after serum treatment. **b** Averages of normalized resistance from different groups were compared after 24 h of serum treatment. Serum from MWCNT-treated mice decreased barrier integrity compared to controls. Marimastat partially recovered barrier integrity in MWCNT exposed group although not back to DM/Veh control serum levels. Data presented are means ± SEM. *p < 0.05 compared to DM/Mar. **p < 0.01 compared to DM/Veh. ***p < 0.0001 compared to DM/Veh and DM/Mar. *p* values based on 2-way ANOVA with Tukey’s multiple comparison test presented for each figure. n = 4 per group

To assess the functional consequences of the MWCNT-generated serum bioactivity, serum from exposed mice was used to assess endothelial barrier integrity changes in an endothelial monolayer using the ECIS assay (Fig. 8). Following treatment with serum from control (DM + Vehicle and DM + Marimastat) mice, mCEC monolayer resistance increased after a brief decline, to ~ 110% of baseline after 1 h and then again to ~ 135% of baseline after 15 h. Treatment with serum from 40 µg/Veh-treated mice significantly reduced barrier integrity acutely and continuously for the ~ 20 h assay, to approximately 70–80% of baseline levels. Serum from mice treated with MWCNT + Marimastat also decreased integrity initially, but cells appeared to recover more quickly than cells exposed to serum from MWCNT treatment alone, although recovery was not fully back to control levels and was not significantly different from MWCNT alone.

## Discussion

**Fig. 9:**
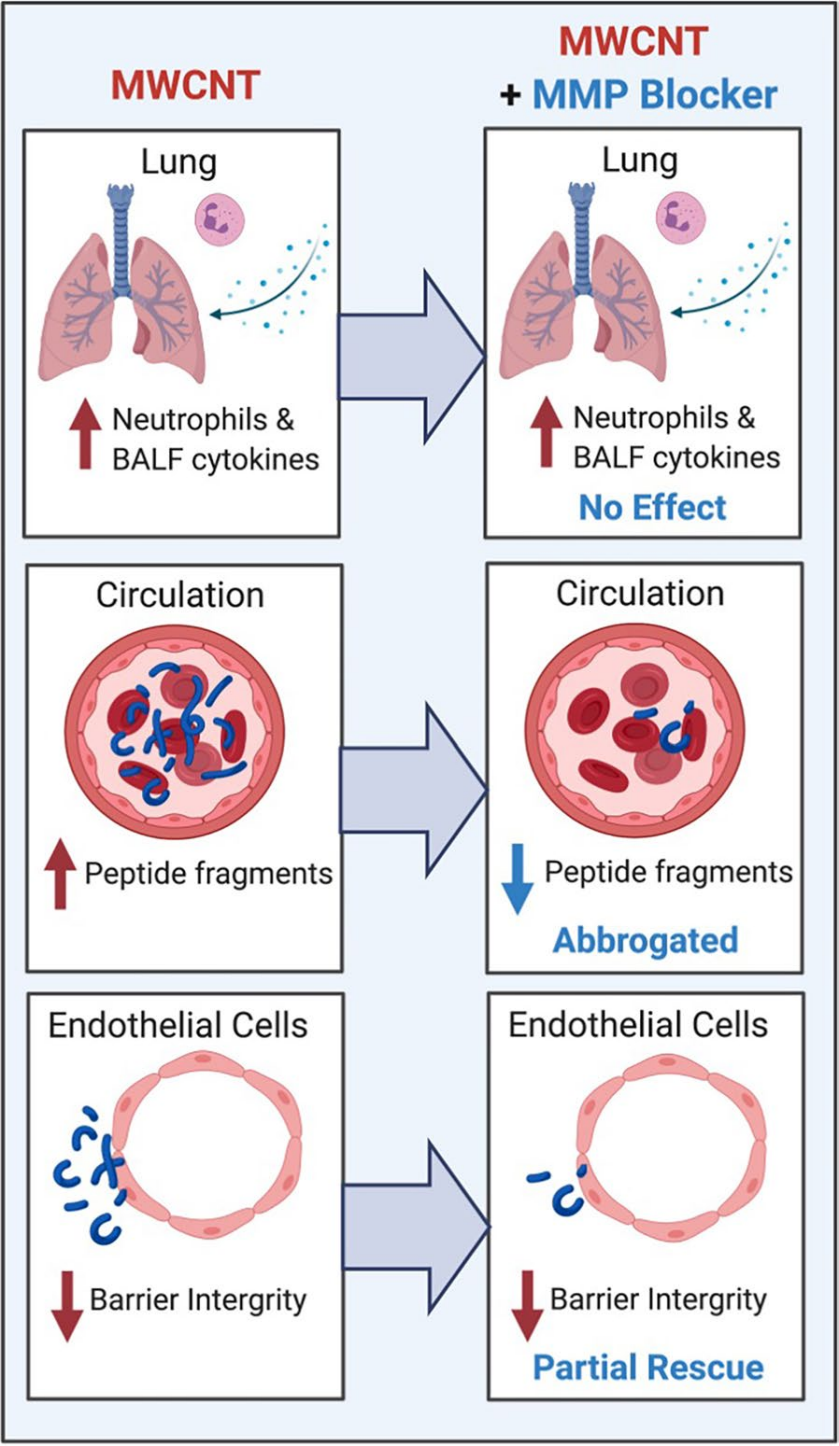
Graphical Summary. Consequences of MWCNT oropharyngeal exposure in C57BL/6 mice. MWCNT induced pulmonary inflammatory activation as evidenced by increased neutrophilia and dose-dependent cytokine BALF profile and whole lung gene expression. MMP blockade did not play a role in the observed pulmonary findings. However, MMP activity was shown in induce serum peptide profile changes in a dose-dependent differential manner. Administration of broad-spectrum MMP inhibitor Marimastat abrogated these serum changes and return peptide profiles to near unexposed levels. The systemic consequences of these serum changes were evaluated in vitro. Serum from MWCNT exposed mice decreased endothelial barrier integrity as assessed via ECIS. Marimastat treatment, presumably through blockade of MMP activity in the lung, resulted in serum with considerably less potential for endothelial cell activation. These findings provide a mechanism for MWCNT action in the lung and starts to elucidate the systemic consequences of inhalation exposures to MWCNT. Additionally, serum findings provide a base for the exploration of MWCNT exposure and pathological systemic consequences, as well as the identification of biomarkers of both

In the present study, we utilized a broad-spectrum MMP inhibitor administered in the lung to demonstrate that: (1) lung MMP activity does not mediate initial MWCNT-induced pulmonary innate inflammation; (2) MWCNT-induced circulating peptides are generated as a result of MMP activity in the lung; and confirms that (3) attendant bioactivity of serum from MWCNT-exposed mice is driven in part by the pulmonary-derived circulating peptides. The pulmonary specific delivery of the MMP inhibitor and acute time frame of the study design, along with the subsequent serum peptide profile changes, infers a pulmonary MMP-mediated mechanism of action for inhaled MWCNT exposures, as has been previously identified with MWCNT and other inhaled pollutants [26, 29] (Fig. 9).

MMPs are elevated in lung pathologies such as pulmonary hypertension, asthma, COPD, pulmonary fibrosis and cancer [42, 43]. Local cells produce MMP-9 when stimulated, and additional generation may occur from immune cells recruited to the lung microenvironment in response to insult; importantly, reactive oxygen species alone can activate constitutive MMPs [44]. Pulmonary oxidative stress is clearly elevated by MWCNT aspiration [45–47]; however, we did not assess the contribution of oxidative stress to the systemic spill-over in this study. The biological pathways underlying nanomaterial-mediated respiratory pathology seem, at least on the surface, to mirror those of air pollution exposures: activation of inflammatory signaling, oxidative stress, enzyme activation and ECM remodeling [47–49]. However the impact of MWCNT inhalation exposure outside the lung, while evident in many studies, remains an incompletely understood phenomenon. The present study therefore suggests that many downstream/extrapulmonary effects of inhaled particulates, such as MWCNT, may be related to pulmonary MMP activation and spillover of fragmented peptides, including matrikines, into the circulation, which in turn drive endothelial inflammatory responses and impair barrier function.

Research on MWCNT-mediated systemic toxicity has generally focused on vascular [50–53] and immune responses [49, 54–57], however gaps remain in our understanding of how toxic effects move beyond the lung. Our previous work showed a role for MMP-9 and endothelial CD36 in a ligand generating/ligand binding relationship in mediating MWCNT-induced vasomotor impairment [26]. Serum from MMP-9 deficient mice exposed to MWCNT failed to recapitulate the dilatory inhibition induced by serum from wild-type mice treated with MWCNT, providing the first evidence of a possible mechanistic role of MMPs in MWCNT-mediated generation of serum bioactivity [26]. Findings by Mandler et al. of a TSP-mediated peripheral microvascular vascular impairment following MWCNT exposure provided further evidence, which narrowed the players in the MWCNT-mediated vascular outcomes [27]. TSP is a widely expressed matrix glycoprotein that has roles as both a substrate of MMP-9 and a ligand for CD36 and CD47. We observed an ~ 50% increase in circulating TSP after MWCNT treatment, but more interestingly we identified a 59-mer peptide fragment containing the thrombospondin repeat domain (the region that interacts with CD36), which was elevated ~ 1000% over control [29]. Since TSP is not only an important modulator of nitric oxide signaling [58], but also an MMP substrate, this protein and related peptide fragments may reflect an important pathway for systemic effects of pulmonary particulate exposures.

Previously, we showed that vascular CD36 was essential for vasorelaxation impairments induced by serum from ozone-exposed mice [59], in addition to repercussions documented from MWCNT-exposed mouse serum [26]. The implication is that pulmonary MMP-derived circulating peptides may act through cell-surface scavenger or pattern recognition receptors to drive systemic pathology. We have previously shown that MWCNT exposure produces overlapping BALF and serum peptide profiles and composition corresponding to, among others, ECM components and MMP substrates [29]. Additionally, MMP-9 protein levels were shown to be elevated in lung tissue and BALF in these early studies. Although we did not assess MMP levels in the present study, the signifcant findings, observed with targeted MMP blockade, points to changes in either protein or activity levels of these proteases. We hypothesized that the hallmark inflammatory activation and frustrated phagocytosis seen with MWCNT exposures may drive elevated and sustained MMP levels that result in not only lung ECM degradation, but barrier integrity impairments that may result in the release of these degradation products into the systemic circulation. The role of MMPs in lung pathologies have been extensively explored and shown to be important in the inflammatory response to exposures as well as in maintaining vascular homeostasis and regulating vascular permeability [21, 60, 61], which might facilitate the transfer of bioactive fragments into the systemic circulation.

With respect to the bioactivity in serum observed from the MWCNT treatment, we have measured comparable bioactivity in terms of endothelial cell activation, as well as vasomotor alterations, with inhalation of various particles and gases at environmentally-relevant concentrations, and notably in human subjects [9, 28, 29, 62–64]. Circulating bioactivity has also been demonstrated to be a hallmark of several inflammatory clinical syndromes, including atherosclerosis and obstructive sleep apnea [38, 39], and exposure such as cigarette smoking [65]. A recent study by Mostovenko et al. also demonstrated that MWCNT exposure induces peptide changes in cerebrospinal fluid and blood of male C57BL/6 mice which corelate with mechanisms invovled in neuroinflamattion, blood brain barrier disruption and hyperexcitation phenotypes, strengthening the idea of adverse systemic consequnces from pulmonary exposures to MWCNT [40]. Interestingly, of the inflammatory markers used in assessing serum inflammatory bioactivity in this study, MMP blockade reduced only *Ccl2* and *Vcam1* gene levels in endothelial cells exposed to MWCNT-exposed mouse serum. This decrease in inflammatory activation in endotherlial was facilitated by the inhibition of CD36 scavenger receptor (Fig. 7a, b). Both *Ccl2* and *Vcam1* are known to play key role in CD36 signaling and recruitment of inflammtory cells to site of exposure [66, 67]. Deficiency in CD36 has been shown to be reduce proinflammatory signaling, cell recruitment, *Ccl2* and *Vcam1* levels in models of ischemic stroke and atherosclerosis [66, 68, 69]. Thus, the reduction in these genes in our study when CD36 is blocked strengthens the possibility of a CD36 role in mediating some MWCNT-induced systemic outcomes via receptor ligands generated by pulmonary MMP activity. Our study adds depth to the existing attempts to identify blood component changes in relation to respiratory exposures, which tend to center on identification of biomarkers of exposure [70, 71] rather than with determining potential systemic impacts of exposure-generated factors.

The findings of serum-borne bioactivity after pulmonary MWCNT treatment must be couched within the limits of the study design. For one, the relevance of bolus dosing by oropharyngeal aspiration in comparison to whole body inhalation continues to be an area of debate. Mercer et al. demonstrated that MWCNT distribution is similar for both methods of exposure, although inflammatory responses are greater with aspiration [72]. In regards to distribution, 1 day after pharyngeal aspiration exposure, 81.6% and 18% of the MWCNT lung burden was in the alveolar and airway compartments, respectively [64]. However, we have previously determined that average inhalable elemental carbon concentrations observed in U.S.-based CNT facilities was approximately 10 µg/m^3^, which would equate to roughly 2 ng/d of nanotube deposition in a mouse [34]. Thus, the doses of 10 and 40 µg MWCNT by oropharyngeal aspiration reflects 13 and 55 years of deposition (19 and 76 when you factor for a 250 or so work days per year), delivered in a single day. Notably much higher carbon particulate exposures can be observed in countries with less stringent regulations [73]. While deposited MWCNT may have a long persistance in the deep lung, we have not carried out studies to confirm that pulmonary inflammation, MMP activity, formation of bioactive peptides, or vascular responses remained elevated long after exposure. These results which would have relevance for human exposures. Particularly in an occupational setting in the manufacture and incorporation of MWCNT into consumer and industrial products.

## Conclusions

In this inhibition study, we show that pulmonary MWCNT-exposure resulted in the generation of circulating peptide fragments with resultant inflammatory bioactivity in vitro and impairment of endothelial barrier integrity is influenced by lung MMPs. MMP inhibition did not appear to limit the severity of pulmonary inflammation in this context, suggesting that systemic circulatory and vascular effects may be secondary to macrophage activation and pulmonary influx of polymorphonuclear neutrophils. Limitations in the study design prohibit broader interpretation of the results as it relates to the role of pulmonary MMPs for chronic systemic outcomes. However, these data add to the existing literature and help to further define the pathological mechanisms underpinning systemic consequences of pulmonary nanomaterial exposures.

## Data Availability

All data related to this study are publicly available upon reasonable request to the corresponding author.

## Abbreviations

ANOVA: Analysis of variance
BALF: Bronchoalveolar lavage fluid
CCL2: C–C motif chemokine ligand-2
CD36: Cluster of differentiation 36
CD47: Cluster of differentiation 47
CNT: Carbon nanotube
COPD: Chronic obstructive pulmonary disease
CT: Cycle threshold
Cxcl1/2: The chemokine (C-X-C motif) ligand 1/2
DMSO: Dimethylsulfoxide
ECIS: Electric cell-substrate impedance sensing
ECM: Extracellular matrix
IACUC: Institutional Animal Care and Use Committees
ICAM1: Intercellular adhesion molecule 1
IFN-γ: Interferon gamma
IgG: Immunoglobulin G
IL-10: Interleukin 10
IL-12p70: Interleukin 12 active heterodimer
IL-1β: Interleukin 1 beta
IL-2: Interleukin 2
IL-4: Interleukin 4
IL-5: Interleukin 5
IL-6: Interleukin 6
KC-GRO: Keratinocyte chemoattractant-human growth-regulated oncogene
Mar: Marimastat
mCEC: Mouse cerebrovascular endothelial cell
MMP: Matrix metallopeptidases
MWCNT: Multi-walled carbon nanotubes
OCT: Optimal cutting temperature
PBS: Phosphate buffered saline
PGP: Pro-gly-pro
PLGS: ProteinLynx Global SERVER™
SCIP: Serum cumulative inflammatory potential
Tbp: TATA-box binding protein
TGF-β1: Transforming growth factor beta 1
TNF-α: Tumor necrosis factor alpha
Tsp-1: Thrombospondin 1
VCAM: Vascular cell adhesion molecule 1
VEGF: Vascular endothelial growth factor
Veh: Vehicle
